# Electrophysiological Correlates of Long-Term * Soto Zen* Meditation

**DOI:** 10.1155/2015/598496

**Published:** 2015-01-06

**Authors:** Henrique Adam Pasquini, Guaraci Ken Tanaka, Luis Fernando Hindi Basile, Bruna Velasques, Mirna Delposo Lozano, Pedro Ribeiro

**Affiliations:** ^1^Laboratory of Psychophysiology, Faculdade da Saúde, UMESP, São Bernardo do Campo, SP, Brazil; ^2^Brain Mapping and Sensory Motor Integration, Institute of Psychiatry of Federal University of Rio de Janeiro (IPUB/UFRJ), Rio de Janeiro, RJ, Brazil; ^3^Division of Neurosurgery, University of São Paulo Medical School, São Paulo, SP, Brazil; ^4^School of Physical Education, Bioscience Department, EEFD/UFRJ, Rio de Janeiro, RJ, Brazil; ^5^Institute of Applied Neuroscience (INA), Rio de Janeiro, RJ, Brazil; ^6^Neurophysiology and Neuropsychology of Attention, Institute of Psychiatry of the Federal University of Rio de Janeiro (IPUB/UFRJ), Rio de Janeiro, RJ, Brazil

## Abstract

This study aimed to verify the electrophysiological correlates of the changes in long-term regular meditators. We use modern techniques of high-resolution electroencephalography applied to slow potentials, power spectra, and potencies related to the events. To obtain encephalographic records, we use an assembly of 128 channels in 31 subjects (17 *Soto Zen* Buddhist meditators). The motivation of this study was to determine whether the induced beta power would present an increase in meditators as well as a decrease in induced theta/beta ratio in absolute and relative values. However, opposite to what we expected, no significant change was found in the beta frequency. In contrast, the main findings of the study were correlations between the frequency of weekly meditation practice and the increased theta induced relative power, increase of induced power ratio (ratio theta/beta), and increase of the ratio of induced relative powers (theta/beta ratio) during our task that featured an “adapted meditation,” suggesting that the meditative state of “mindfulness” is much more related to the permittivity of “distractions” by the meditators, with a deliberate reduction of attention.

## 1. Introduction

Attention may be one of the most ancient theoretical constructs of psychology. Meditation, on the other hand, has been considered in western culture as a set of techniques, which strive to train attention focus. The aim of this study was to verify whether the induced beta band power would increase in meditation practitioners. Induced beta band power has been considered as a direct electrophysiological correlate of attention in our studies [1–3]. Travis [4] shows a beta band power increase in the frontal cortex during transcendental meditation practice, relating this finding to an active attention processing. In another related study, a significant beta power increase is verified during the meditative state compared to the premeditative state (only sitting and relaxing) in* yoga* practitioners, highlighting a more vigorous brain activity during the meditative process. Saggar [5], on the other hand, found a reduction in beta power in the parietal and occipital regions, during meditation with the attention focused on breathing. Gamma waves, which have been considered by some as a physiological* continuum* of beta waves [2], also showed an alteration, which was associated with the meditation practice. In their study, Lutz et al. [6] observed a high level of gamma waves in Tibetan Buddhist monks with great meditation experience, already in the premeditative state, compared to the control group, and such waves kept increasing as the meditation practice advanced, suggesting that attention is a flexible skill, which can be trained [6]. Cahn et al. [7] also found an increase in the gamma oscillations in the occipital region of experienced meditators. However, some authors point out that gamma activity can be confused with muscular artifacts, that is, with EEG contamination by muscular activity [2, 8].

The hypothesis of this study anticipated that the attention electrophysiological correlates would be gradually modified by the* Soto Zen* meditation practice, classified as a full attention meditative technique [9].

## 2. Methodology

### 2.1. Subjects

Thirty-one healthy adults participated in this research (9 women and 22 men), with ages ranging between 19 and 66 years (mean = 40, SD = ±11.99), including both women (29%) and men (71%), with no visual or auditory problems, no neuropsychiatric disorders, and no history of drug and alcohol abuse. Fourteen subjects (45.20%) were nonmeditators, counting a majority of men (78.57%), with an average age of 34.64 years (SD = ±10.52). Seventeen subjects (54.8%) were actual habitual meditators, counting a majority of men (64.71%), with average age of 44.61 years (SD = ±11.56), including Buddhist* Zen *monks and practitioners with at least two years of experience and with minimum training frequency of three times a week (mean = 4.41, SD = ±1.62), with average proficiency meditation time of 1429,88 hours (SD = ±1772.41) (Table 1). All subjects from this sample received and signed the informed consent form (ICF), in agreement with the Brazilian Law CNS 196/96 from the Brazilian Ministry of Health, approved by the university (Ethics Committee of the* Universidade Metodista de São Paulo*, UMESP).

### 2.2. Stimuli and Tasks

The utilized tasks have become standardized in our laboratory, as they have been used in previous studies [2, 10]. A commercial computer program (Stim, Neurosoft Inc.) controlled all aspects of the tasks. In the sustained attention task, the visual stimuli forming the pairs “stimulus-target” (S1-S2) consisted of small rectangles (eccentricity ±0.8°, S1: 100 ms of duration, S2: 17 ms; on white background). During half the task, the rectangles had a grey circle—the task target—with an eccentricity of ±0.3°. S1 was followed by S2, with 1.6-second intervals.

The subjects were told that the first rectangle (stimulus, S1) would appear on the computer monitor warning that it would appear again after 1.6 seconds but that it would be faster and containing or not the target circle (target, S2). The subjects had to decide whether a target circle existed or not inside the second rectangle (S2), and they had to indicate the presence of the target by pressing with the right thumb the right button of a device they were holding, which was similar to a videogame, or they had to indicate the absence of the target by pressing the left button of the device. We did not underline the reaction time during the instructions on purpose, and we measured the performance considering exclusively the percentage of correct tests from the experiment total of 96 tests.

The passive observation task consisted of the same number of tests, but there was no need to answer for S2. The subjects were instructed to keep their eyes fixed on the middle of the monitor and to focus the attention on the breathing, as in a kind of “adapted meditation.” With such instructions, we standardized the same methodology to obtain a “meditative state” for all subjects and even those without any proficiency in meditation obtained a proficiency of a few minutes by the end of the experiment.

### 2.3. EEG Record

We used a fast positioning system for electrodes Ag/AgCl, consisting of an extended 10–20 (128-channel system), essentially with 3 lines of electrodes between the frontal, central, and occipital sets, one line between frontal and FP line, and two lines beyond (below) the occipital electrodes line (Quik-Cell, Quick-Neuromedical Supplies), and an impedance-reducing saline solution, so that only the reference and ground electrodes would need abrasion. We were very careful about keeping the impedance below 5 k Ohms, and the unstable channels were excluded from the analysis. During the three stages of the experiment, data were collected through two DC amplifiers, each one of them containing 64 channels (Synamps, Neuroscan Inc.), and the initial data processing (before the average calculation) was conducted with the help of the Scan 4.3 software plan.

Finally, we analyzed data through the Scan 4.3 software and through the Curry V 6 commercial software (Neurosoft Inc.). The EEG was collected continuously, and the task-related epochs were limited to an interval of 300 ms before S1 to 400 ms after S2 in the passive observation of the stimuli and sustained attention tasks and to an interval of 2400 ms before and 200 ms after the subject pressed the button in the reasoning task. The baseline was defined starting from 300 ms for all epochs. The epoch exclusion was done visually and therefore automatically, for the eye movements and muscular contractions. The visual inspection was used to eliminate epochs containing other artifacts, such as broken channels and cable movements. Isolated electrodes exhibiting frequent electronic noises were also visually excluded.

### 2.4. Obtained Electrophysiological Correlates

The electrophysiological correlates obtained from both tasks (passive observation and sustained attention) included the analysis of the band power variations in the frequencies of interest (theta, alpha, and beta) and the simple analysis of the power spectrum. All cortical region was used as a form of analysis consisting of the computation of a spectral average from all segments of EEG electrodes (overall strength field and overall power field) [10] with the objective of obtaining a waveform which quantifies the average activity recorded between all electrodes distributed on the scalp [11] without focusing on a specific area. This conversion is important because it allows a global analysis of all electrodes and allows the identification of important peaks of power in any region of the scalp, demonstrating the idiosyncratic cortical activation for each individual while performing tasks demanding cognitive effort [1, 2].

Also according to Basile et al. [1], cortical electrical activity associated with attention is characterized by multifocal activity and complex distribution and is highly variable among individuals; it is possible that the complexity and the number of possible functional corticocortical pathways excited are enough to allow the formation of a set of interconnected cortical areas that are variable between individuals, before and during the execution of any given task, concluding that the radical idea of fixed and universal functions cannot be attributed to non-sensorimotor areas between individuals.

The study of the* event-related band power* analysis shows two elements, which include the* induced band power*, not synchronized with the events, and* evoked band power*, synchronized with the events [12]. Such analysis method allows for the mapping of a certain signal within a function, which depends on time and frequency just like a sliding window on the temporal axis; that is, this measure corresponds to the power of the oscillatory activity average within certain frequency band of interest [12], by limiting the signal to be analyzed in such a way that this could be considered to be stationary, in order to conduct a satisfactory analysis through the Fourier transform [13].

**Figure 1:**
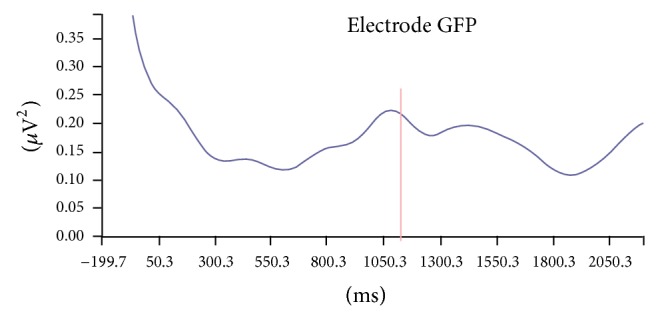
Power oscillation study for predetermined bands (beta band power, 20 Hz)—induced band power. A band power peak of 0.222* μ*V^2^ is found, occurring 1087 ms after the appearance of the first stimulus (S1)—induced band power analysis.

First, we performed a band power analysis in the frequencies of interest, for the theta (centered at 5 Hz), alpha (centered at 10 Hz), and beta (centered at 18, 20, and 25 Hz) frequency bands, computing both their evoked (stimulus-locked) and their induced components, since there is evidence that such element reflects more complex cognitive processes, while the evoked component mainly reflects stimulus-oriented processes [14]. The induced component was obtained by recording the band power peak in a time span of 500 ms to 1600 ms after the appearance of S1 (expectation period for S2) (Figure 1).

**Figure 2:**
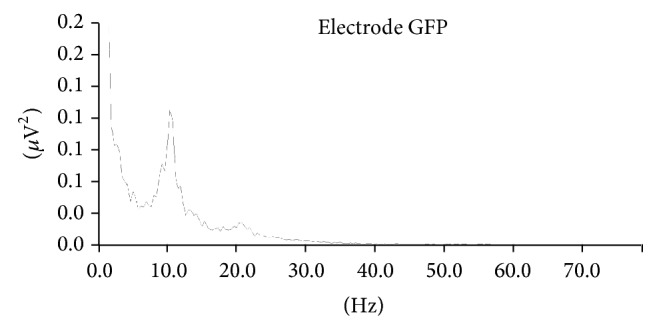
“Band power spectrums” study in the “frequency domain” (simple band power). The figure shows band power peaks for two bands of interest: alpha (frequency 10.38 Hz and 0.109* μ*V^2^ of power) and beta (frequency 20.76 Hz and 0.018* μ*V^2^ of power).

In our band power spectrum analysis within frequency (Figure 2), we analyzed the theta power peaks (3–8 Hz), because this band is related to a more disperse way of allocating attention, without any focus [15]; the alpha power peaks (8–12 Hz), for the fact of this band power being generally associated with the meditative state [16]; and the beta power peaks (13–30 Hz) together with its three frequency subbands: beta 1 (14–17 Hz), beta 2 (17–23 Hz), and beta 3 (23–30 Hz), under the experimental conditions, since these frequency bands are directly related to focused attention [1, 3].

## 3. Results

The main hypothesis of this research anticipated that attention and its electrophysiological correlates would be gradually modified by the habitual meditation practice. Taking the independent variable “time of meditative practice” as our basis, we applied the Shapiro-Wilk test and verified that distribution was not normal. Therefore, we used the Spearman  *ρ* statistical test, to verify the existence of statistically significant correlates, and the Mann-Whitney statistical test, to verify the existence of statistically significant differences between the groups (meditators and nonmeditators).

The “time of meditative practice” (calculated in hours of meditation), as well as the “practice weekly frequency,” was correlated with the electrophysiological correlates in the three different conditions studied. The electrophysiological correlates analyzed were, mainly, the absolute measures of the theta, alpha, and beta band power peaks (the respective frequencies where the peaks occurred—peak frequencies—were also recorded) and the theta, alpha, and beta induced band powers (established at 5 Hz for theta, 10 Hz for alpha, and 18, 20, and 25 Hz for beta). We also correlated the meditation practice time with a series of relative ratios and measures of the induced band powers.

Significant positive correlations were found between the “meditation practice weekly frequency” and the ratio for the theta (5 Hz) and beta (theta/beta) event-related band powers in the passive observation of the stimuli task for beta, fixed at a frequency of 18 Hz (*ρ* = 0.36; *p* = 0.048) and 25 Hz (*ρ* = 0.36; *p* = 0.049). Significant positive correlations were also found between the “meditation weekly frequency” and the relative induced theta band power in the passive observation of the stimuli task (*ρ* = 0.42; *p* = 0.02) and between the “meditation weekly frequency” and the theta/beta ratio of the relative induced band powers in the same passive observation of the stimuli task (*ρ* = 0.43; *p* = 0.016) (Table 2).

Significant negative correlations were found between the alpha peak frequency in the focused attention task and the “meditation practice time” (*ρ* = −0.52; *p* = 0.003) and the “meditation weekly frequency” (calculated as days per week) (*ρ* = −0.41; *p* = 0.021).

## 4. Discussion

In this study, the electrophysiological correlations were associated with the “frequency of weekly meditation practice” (FWMP) analyzing their absolute and relative measures and their ratios. A finding of this study was the correlation between the meditative practice and the decrease in the alpha peak frequency. Although the decrease in the alpha peak frequency induced by meditation has already been proved in the literature [7, 16–18] and even has been considered a signature of experienced meditators [5], we prefer to be cautious and look for corroboration from future studies. Positive and significant correlations were found between the FWMP and the ratio of theta power (5 Hz) and beta induced (event-related band power) in the task of passive attention when beta was set at 18 Hz (5 Hz theta/beta 18 Hz) (*ρ* = −0.36; *p* = 0.048) and 25 Hz (theta 5 Hz/25 Hz beta) (*ρ* = 0.36; *p* = 0.049). To obtain the theta/beta ratio we used a theoretical framework by the paradigm study related to electrophysiological correlates of attention deficit hyperactivity disorder (ADHD) [19–21].

Therefore, positive and significant correlations were also found between FWMP and theta relative power in their induced component (induced band power) in the task of passive observation of stimuli (*ρ* = 0.42; *p* = 0.02). To calculate the relative power, a basic calculation was used which divides the peak value of the power of interest by the sum of all the peak values of the powers recorded [22, 23]. Thus, to calculate, for example, the relative power in induced theta component, the induced theta peak power was divided by the sum of the peak values of the powers induced theta, alpha, and beta (i.e., theta/(theta + alpha + beta)).

Then, after obtaining the theta and beta relative power (evoked and induced components) we decided to calculate the ratio of these relative powers, also following the previous paradigm to the ADHD study, and found a significant positive correlation between FWMP and theta/beta ratio of induced relative powers (*ρ* = 0.43; *p* = 0.016), confirming the findings presented above associated with theta/beta induced powers presented in absolute values ratio. Furthermore, we have noticed that studies of powers induced-events (induced band power, IBP) are more difficult to interpret due to, seemingly, being missing in the current literature. The increase in induced theta power in frontal regions may be related to a state referred to as mind wandering (spontaneous mental activity based on their introspective experience) [24, 25].

According to Aftanas and Golocheikine [26] the event-related power (event-related band power, EBP) associated with its induced component (induced band power, IBP) is usually related to an increase in neuronal excitation, such as that observed during the increase in cognitive activity, reflecting an externalized attention which appears in higher vigilance and anticipation states. There is evidence that the induced component reflects more complex cognitive processes such as those involved in attentional processes, while the evoked component reflects mainly processes driven by stimuli [14]. The induced theta power increases in a wide variety of tasks, and it is a plausible assumption that this power is related, at least in part, to nonspecific factors related to the task, such as attentional demands, and difficulty performing cognitive load, suggesting that theta rhythm is generated by various brain structures of spontaneous form or related to events [27].

By the way, theta oscillations can be seen as a putative correlate attentional expectation [1]; it is currently postulated that theta oscillations reflect a “more general brain integrative mechanism” instead of a specific focused attention integrative mechanism and memory processes [15]. Theta waves have been associated with decreased sustained attention to tasks and related to different stages of transition from wakefulness to sleep [28] supporting the hypothesis that meditators seem to maintain their state of brain activity at a level transition between full wakefulness and early sleep stages [29, 30]. Aftanas and Golocheikine [26] and Kubota et al. [31] also observed an increase in theta power in meditators; however they used absolute power and were limited to the frontal midline. With regard to induced beta power, Basile et al. [1], in an experiment S1-S2, the same model used in this working model, found that the induced beta power increased in the period between S1 and S2, with its peak just before S2, supporting the hypothesis that this frequency range and the slow powers are the most direct correlates of attention [1, 3].

A significant amount of analysis can be performed with the use of quantitative electroencephalography applied to absolute, relative measurements and their ratios between the measures. The relative measurements are usually calculated and presented together with the absolute measurements giving a contribution to a more accurate analysis of the data [22]. Also according to Pivik [22] many things can contribute to power variation in a given region of the skull, such as muscle artifacts and the thickness of the skull; therefore, obtaining and comparing powers ratios are presented as a promising method of study.

We have not been able to find much discussion using ratios between theta and beta induced powers (induced band power), such as the study of the analysis of power in predefined tracks (event-related band power) taking into account its induced component [24, 25]. Nevertheless studies correlating theta/beta ratios and attention using simple analysis of the spectra power are being widely used in the researches on ADHD [19]. Therefore, integrating our findings with studies that interpret the induced theta and beta power in their absolute values outlined above, taking for granted that the induced component reflects more complex cognitive processes (e.g., those involved in attentional processes) [14], and considering that theta/beta ratio reflects the functionality of attentional processing of the cortical substrates [20], such as neurofeedback traditional protocols that call for the reduction of inattention and impulsiveness, is directly related to the increased beta and decreased theta activity [21].

Thus, we suggest that the meditative state of “mindfulness” technique proposed by Buddhist meditation studied is more related to the development of a “brain more general integrative mechanism” rather than a specific mechanism of integrative focused attention and memory processes [15, 32], since the theta/beta induced ratio power (absolute values), the relative theta power, and theta/beta relative ratio power (also in its induced component) were significantly correlated with the FWMP; as if assiduous practitioner had allowed himself, deliberately, innumerable “distractions” during practice; allowing thoughts, feelings, or sensations arise without sticking to any of them [33]; corroborating the interpretation of “right mindfulness” as the seventh via the “Noble Path” of Buddhism that means “being aware of what is occurring at the present time watching all the thoughts and feelings as they sungem without clinging to them” opposing the focused attention called “right concentration” defined as the ability to focus, “the mind's ability to stand firm on an object” [34–36].

The motivation of this study was to determine whether beta power would present increase in meditators. However, opposite to our expectations, no significant change in the beta frequency band was found. We assume that the beta frequency, especially in its induced component, is presented as a direct correlate of sustained attention [1, 3] and the Soto Zen meditation regarded a technique of “mindfulness” and is an important technique for the control of attentional training processes as a focus training [37–39]. However, during the development of this work we found that the “mindfulness”—“right attention” is the seventh track from the “Noble Path” of Buddhism—is presented as a construct that is more related to the permittivity of “distractions” by meditators with an intentional reduction of sustained attention and not an effort to focus and select a stimulus to be processed that corresponds to the Buddhist term known as “right” concentration which is the eighth pathway “Noble way.”

Among the arguable points highlighted by our research, the most significant may be the lack of standardization of the methodology adopted in the researches about meditation.

Meditation can be divided into two groups, “full attention meditation” and “focused attention meditation,” depending on how the attention processes are directed [17, 39]. However, it is hard to classify a certain meditative practice as being “purely full attention” or “purely focused attention,” since the majority of meditation techniques are found to be somewhere between these two poles [40]. Also, knowing for sure under which condition the practitioner is at the moment of the electroencephalographic records is a methodological problem, since a specific frequency band was attributed to each meditation category: the techniques based on the “focused attention” are characterized, mainly, by the beta and gamma activity, while the “full attention” techniques are determined by the theta activity [8].

One more question observed in the studies about meditation is related to the different attention conditions under which the data are collected among the groups; many times, the control group is given the orientation to simply relax, while the experimental group, composed by the meditators, is instructed to develop its respective meditation techniques [6, 41], establishing different conditions between the groups to be compared.

In conclusion, in order to establish standardization in our study, all subjects, meditators and nonmeditators, executed the same tasks during the data collection period. During the meditative period, we opted for an “adapted meditation,” which represented our passive observation of the stimuli task focusing on breathing, and the data were analyzed after calculating the global field power, respecting the idiosyncrasy in the cortical activation of each individual.

## 5. Conclusion

Our findings suggest that full attention meditation practice is a more generic integrative brain mechanism, rather than a focused attention and memory processing specific integrative mechanism [15], as if the individual who assiduously practices meditation would allow himself or herself, deliberately, to have many “distractions” during the practice, making an effort to not cling to any of them.

As future perspectives, we intend to research the relation between attention electrophysiological correlates and the “focused attention” meditation practice (where we expect to see some relaxation, focus, and rare periods of allowing for distractions from the subjects); we also intend to research the labor activity of professionals who need to maintain the attention focus as much as possible, in addition to trying blocking the distracting factors, such as snipers, some industry workers, and helicopter pilots (all activities where we expect to see an absence of relaxation, intense focus, and absence of allowing for distractions from the subjects). We hope that the study of such states, which have the potential to modify attention and its respective electrophysiological correlates, would bring more information about the neurophysiology involved in cognitive activities with important attention demand.

## Figures and Tables

**Table 1:** Participants data (*n* = 31).

Variable	Category	*n*	%	Average	Standard deviation
Participants	Meditators	17	84.7		
	Nonmeditators	14	15.3		

Gender	Meditators				
	Men	11	64.71		
	Women	06	35.29		
	Nonmeditators				
	Men	11	78.57		
	Women	03	21.43		

Age	Meditators			44.61	±11.56
	Nonmeditators			34.64	±10.52

Meditation time (approximated hours)	Meditators			1429.88	±1772.41

**Table 2:** Correlation index (Spearman ρ) followed by the measure precision index (*p*), relative values and ratio correlations, meditation weekly frequency versus theta/beta ratio of induced band powers, theta relative induced band power, theta/beta ratio of the relative induced band powers, and alpha frequency peak (all values were collected during the passive observation task).

Variable	EBP induced theta/beta ratio 18 Hz Pas	EBP induced theta/beta ratio 25 Hz Pas	Relative induced theta Pas	Relative induced theta/beta ratio Pas	Alpha frequency Att.
Meditation weekly frequency	0.36^*^ (*p* = 0.048)	0.36^*^ (*p* = 0.049)	0.42^*^ (*p* = 0.02)	0.43^*^ (*p* = 0.016)	−0.41^*^ (*p* = 0.021)
